# Non-canonical helical transitions and conformational switching are associated with characteristic flexibility and disorder indices in TRP and Kv channels

**DOI:** 10.1080/19336950.2023.2212349

**Published:** 2023-05-17

**Authors:** Abigail García-Morales, Daniel Balleza

**Affiliations:** Unidad de Investigación y desarrollo en Alimentos, Instituto Tecnológico de Veracruz. Tecnológico Nacional de México, Veracruz, MEXICO

**Keywords:** Intrinsic flexibility, protein disorder, structural discrepancy, conformational rearrangements

## Abstract

Structural evidence and much experimental data have demonstrated the presence of non-canonical helical substructures (π and 3_10_) in regions of great functional relevance both in TRP as in Kv channels. Through an exhaustive compositional analysis of the sequences underlying these substructures, we find that each of them is associated with characteristic local flexibility profiles, which in turn are implicated in significant conformational rearrangements and interactions with specific ligands. We found that α-to-π helical transitions are associated with patterns of local rigidity whereas α-to-3_10_ transitions are mainly leagued with high local flexibility profiles. We also study the relationship between flexibility and protein disorder in the transmembrane domain of these proteins. By contrasting these two parameters, we located regions showing a sort of structural discrepancy between these similar but not identical protein attributes. Notably, these regions are presumably implicated in important conformational rearrangements during the gating in those channels. In that sense, finding these regions where flexibility and disorder are not proportional allows us to detect regions with potential functional dynamism. From this point of view, we highlighted some conformational rearrangements that occur during ligand binding events, the compaction, and refolding of the outer pore loops in several TRP channels, as well as the well-known S4 motion in Kv channels.

## Introduction

Membrane proteins are dynamic molecular entities whose conformational changes are closely linked to their interaction with lipids, which in many aspects determine their biological functions. These changes are controlled by multiple variables such as temperature, lipid composition, salinity conditions of the medium, pH, binding of chemical ligands, or the electro-mechanical stimuli through the membrane. However, considering the protein intrinsic flexibility at the atomic level also contributes to revealing dynamic information relevant to a better understanding of the associated conformational changes since atoms are in constant motion [1]. At the structural level, the conformational changes that a protein undergoes involve the deformability of the three main secondary elements, *i.e*. α-helices, β-sheets, and disordered loops. The study of deformability in helicoidal structures is particularly significant in membrane proteins since these elements’ size, geometry, and charge distributions are important in terms of flexibility and molecular dynamics [2]. In consequence, flexible helices may have pivotal functional roles in transmembrane segments of membrane proteins, particularly in transporters and channels. On the other hand, the role of intrinsically disordered regions in proteins could also be critical promoting conformational heterogeneity [3].

To open the gate, potassium channels have evolved to have a highly conserved Gly residue, followed by the PXP motif in the middle of segment S6 (the inner pore). The so-called Gly-hinge is used during the voltage-dependent opening of the activation gate [4]. Given the absence of a side group of the Gly, as well as the rigid cyclic structure of the Pro residue, the C-terminal part of the S6 helix is an exceptionally flexible element in those channels [4,5]. This flexibility determines that the region near the PXP motif transits to different conformational states, specifically the 3_**10**_ configuration and the concomitant motion of 6 Å during the pore aperture [6]. In voltage-dependent channels, this mechanism of activation depends on the electro-mechanical displacement of segments S4/S4-S5L as a response to changes in the membrane potential, coupling that motion to the activation gate located at the inner part of segment S6 [7]. In transient receptor potential (TRP) channels, nevertheless, the analog mechanism seems to be a little bit different. Although some representatives of this superfamily of ion channels have been described as slightly voltage-dependent (in TRPV1, TRPM5 or TRPM8, for example) [8,9], they are also susceptible to distinct stimuli, notably chemical ligands, and temperature changes [10]. In this regard, evolution has dictated a mechanism where ligand/temperature activation induces α-to-π secondary transitions in their analogous pore-lining S6 helices which depend on the functional state of the channel [11]. Sobolevsky’s group first reported this transition in the human TRPV6 channel as part of the iris-like opening of this protein [12]. Since this finding, numerous reports have confirmed this observation in other TRP channels, in addition to the discovery of 3_**10**_ helices in these channels, such that practically most members of the TRP superfamily and some Kv and Nav channels possess one (or both) of these structural elements [13,14]. This observation is very relevant in terms of evolution since, in comparison with Kv channels, TRP channels have surely diverged much more recently in life evolution.

To better understand the nature of conformational changes in proteins, it is necessary to study the processes of deformability of the involved secondary structures, as this parameter depends on flexibility in secondary structures. The most stable helical structure is the canonical α-helix (3.6_**13**_-helix), whereas transitions to π- or 3_**10**_-helices are associated with different flexibility degrees, being 3_**10**_-helices transitions considered as processes of the unfolding of α-helices and, intuitively, highly dependent on local flexibility. On the other hand, transitions to π-helices could be considered as the “superfolding” of an α-helix, which also depend on flexibility but probably to a lesser extent than in the previous case [15,16]. Thus, in structural terms, the most distinctive feature of 3_**10**_-helices is their long C**α**_***i***_-C**α**_***i***-**4**_ /C**α**_***i***_-C**α**_***i*****−5**_ distances (more than 1.0 Å/residue); whereas a typical π-helix becomes more compact with a shortening of those distances, and a concomitant widening of the resulting turns [17]. Besides these structural aspects, some sequence patterns have been described as folding/unfolding triggers in non-canonical helices. Some of the residues that seem to favor π-superfolding are those with large aromatic or bulky hydrophobic side-chains, as well as Asn which often preceded proline residues [18]. 3_**10**_-helices, in turn, are generally favored with β-branched side-chains N-terminal to proline residues, as well as Asp and aromatic residues close to glycines [17,19]. Regardless of these observations, a characteristic π- or 3_**10**_-sequence motif has not been described so far, and the prevalence of certain residues in these non-canonical structures seems rather to indicate that they must depend on the physico-chemical context of each protein. In any case, it has been proposed that any of the two configurations could be interconvertible forms of the most stable structure of all, the α-helix [16].

To improve our understanding of helical folding and try to establish predictive rules relating to primary sequence and secondary structure, herein we studied the relationship between amino-acid composition, side-chain flexibility, protein disorder, and the propensity to facilitate non-canonical helicoidal transitions in TRP and Kv channels whose molecular structure is known. Our main proposal intends to determine if the known interconvertibility reported between the helical structures in Kv and TRP channels depends on the intrinsic flexibility of the segment in question. The results presented here indicate that those sequences that transit to π-helices are frequently related to low flexibility profiles (*i.e*., they are rigid and ordered regions in terms of their normalized B-factors). On the other hand, transitions to 3_**10**_-helices are facilitated when the amino acid sequence has a composition whose average flexibility is relatively higher (*i.e*., they are flexible regions, although also ordered), at least for the channels studied herein. Likewise, we describe several cases where these structural elements could be key in the recognition of specific ligands. We have also noticed that both side-chain flexibility and protein disorder are not equivalent concepts but complementary and that a disordered sequence is not necessarily always flexible, and vice-versa. The correct interpretation of these two parameters could facilitate the interpretation of the conformational heterogeneity exhibited by these proteins. Thus, our findings may serve to establish a basis for studying conformational flexibility in membrane proteins and to visualize a predictive theoretical framework for a better understanding of the dynamical gating process in these ion channels.

## Material and Methods

### Structural search criteria

To carry out this study, we studied 38 proteins where non-canonical helical transitions (*i.e*. α-to-π and α-to-3_**10**_) have been described. These proteins encompass the whole TRP channel superfamily (TRPV1–6; TRPM1–8; TRPC1–7; TRPML1–3; TRPP1–2; TRPA1; TRPN1 and TRP1) as well as 8 members of the Kv, Nav, and HCN channel families, including the Shaker K-channel, the Kv1.2 (Shaker-like) channel, the Kv1.2/2.1 *paddle* chimera, the KCNQ1 (Kv7 .1), hERG (Kv11.1) and HCN1 channels, as well as the bacterial cyclic nucleotide – regulated K-channel (MloK1) and the bacterial voltage-gated Na**+** (Nav) channel, NaChBac.

### Sequence dataset and PDB files

The selected sequences were integrated into a database for all queries: (1) TRPV1 from rat (NP_114188.1) (PDB codes: 5irz, 5irx, 7lpa, 7lpb, 7rqu, 7rqx, 7rqz); (2) TRPV2 from rat (Q9WUD2) (6bo4, 6bo5, 6oo5, 6oo7, 6u84, 6u86, 6u8a, 6u88); (3) TRPV3 from *Homo sapiens* (NP_659505.1) (6mho, 6mhw) and mouse (AAM33069) (6lgp, 6dvw, 6dvy, 7ras, 7rau); (4) TRPV4 from *H. sapiens* (NP_001170902.1) (7aa5) and *Xenopus tropicalis* (XP_002932129.1) (6bbj); (5) TRPV5 from rabbit (Q9×SM3.1) (6b5v); (6) human TRPV6 (6BO8_1) (6bo8, 7s8c); (7) human TRPM1 (Q7Z4N2.2); (8) TRPM2 from *Nematostella vectensis* (A7T1N0.1) (6co7), zebrafish (6D73_A) (6d73, 6pkv, 6pkw, 6pkx), and human (NP_003298.2) (6mix, 6miz, 6mj2); (9) human TRPM3 (NP_066003.3); (10) TRPM4 from mouse (AAH96475.1) and human (NP_060106.2) (6bqr, 6bqv); (11) TRPM5 from *H. sapiens* (NP_055370.1); (12) TRPM6 also from human (Q9BX84.2); (13) TRPM7 from mouse (Q923J1.1); (14) TRPM8 from *Parus major* (6O6A_A) (6o6a, 6o6r, 6o72, 6o77), *Ficedula albicollis* (6NR2) (6bpq, 6nr2, 6nr3, 6nr4), and mouse (NP_599013.1) (7wra); (15) human TRPC1 (EAW78963.1); (16) TRPC2 from mouse (EDL16568.1); (17) human TRPC3 (Q13507.4); (18) TRPC4 from *Danio rerio* (NP_001276810.1) and mouse (Q9QUQ5.1) (5z96); (19) mouse TRPC5 (Q9QX29.2); (20) human TRPC6 (AAH93660.1) and (21) TRPC7 (AAI28186.1); (22) TRPML1 (Q99J21.1) and (23) TRPML2 (Q8K595.1) from mouse; (24) human TRPML3 (Q8TDD5.1) and from *Callithrix jacchus* (F6RG56.1) (5w3s); (25) human TRPP1 (NP_000288.1) (5k47); (26) mouse TRPP2 (A2A259.1); (27) human TRPA1 (NP_015628.2) (3j9p, 6pqq, 6pqo, 6pqp); (28) TRPN1 from *Drosophila melanogaster* (5vkq); (29) TRP1 from *Chlamydomonas reinhardtii* (6pw5); (30) Kv1.2–2.1 *paddle* chimera from rat (2R9R_H) (2r9r); (31) Shaker-IR (7sip), and its isoform E from *Drosophila* (NP_728123.1); (32) the Kv1.2 channel from rat (2A79_B) (2a79); (33) human KCNQ1 channel (NP_000209.2) (6uzz, 6v00, 6v01); (34) hERG (Q12809.1) (5va1, 5va2, 5va3); (35) HCN1 (NP_066550.2) (6uqf, 5u6o), (36) spHCN from *Strongylocentrotus purpuratus* (NP_999729.1); (37) MloK1 (WP_010911524.1) (4chv), and (38) NaChBac (6vwx). All structural analyzes were generated, processed and visualized using the PyMOL Molecular Graphics System, Version 1.2, (Schrödinger, LLC), and the ChimeraX package.

### Multiple sequence alignment and logo generation

Multiple sequence alignments were performed using the ClustalW informatic tool [20], incorporated into the Bioedit free software (https://bioedit.software.informer.com/). The percentage of identity was calculated using the SIM resource algorithm (https://www.expasy.org/resources/sim). Once the sequence alignments were generated, the corresponding logos were built using the Weblogo 3.0 server [21].

### Flexibility profiles generation

As it has been demonstrated, flexible regions in proteins can be predicted from the primary sequence through the evaluation of the normalized B-factors [22]. Thus, we have developed a bioinformatic tool based on estimations of the intrinsic flexibilities for each amino acid [23,24]. Basically, our FlexiProt algorithm predicts the local side-chain flexibility, which correlates the composition of amino acids in a protein sequence in the context of the *N*- and C-terminal neighbors for each residue coded in the primary sequence. The program assigns a weighted normalized B-factor value based on a stiffness classification, according to structural aspects associated with the theoretical degrees of freedom for each of the 20 side-chains [25,26]. Thus, normalized B-factors are clear indication of the local residue flexibility according to the next expression:

(1) $$nBf=\frac{B-Bm}{B\sigma }$$

where the B-factor normalization (*nBf*) depends on the atomic thermal factor reported on the PDB (*B*), the sample mean value of B-factors (*Bm*) for a dataset of protein structures, and the standard deviation of the sample distribution of such thermal factors (*B*σ) for a determined structure [27]. Therefore, our algorithm quantifies a characteristic (*i.e*. intrinsic) flexibility for each residue, based only on the primary sequence, regardless of the experimental conditions used to solve any protein. With this information, each sequence can graphically generate a flexibility profile as a (*x,y*) plot, which is analyzed segment by segment according to structural or dynamic criteria.

Here, we focus our analysis on the whole transmembrane domain (S1–S6) in selected TRP and Kv channels. To obtain an estimate of the relative flexibility of a given segment, these flexibility profiles are then mathematically normalized and averaged, showing the inverse of the mean B-factors for the segment studied (the normalized mean B-factor, *mBf*, equation 2) and comparing this value with its structural equivalent in another part of the same protein or in a distinct protein.

(2) $$mBf=-\frac{1}{n}\overset{n}{\underset{i=1}{\sum }}nBf$$

### Prediction of protein disorder

The prediction of disordered regions was calculated using the PrDOS algorithm. This predictor is based on a comparative analysis of primary sequences where differences in conservation patterns of length, location, and composition of intrinsically disordered regions (IDRs) are highlighted, using machine learning techniques to detect them [28]. Hence, this predictor of protein disorder distinguishes stretches showing a trend to be autonomously folded versus those sequences with more propensity to exhibit spatial fluctuations [29]. The PrDOS online server (https://prdos.hgc.jp/cgi-bin/top.cgi) is available to all users and it consists of two predictors: one based on local amino acid sequence information and the second based on structural comparisons with template proteins deposited in the PDB. Each target amino acid sequence is converted to a position-specific scoring matrix (PSSM) and two predictions are made based on the local amino acid sequence information. With this information, the PrDOS predictor uses the alignments of sequences of interest with known structures. Finally, the results of these two predictors are combined and the weighted average between them is then calculated, resulting in a binary verdict within a confidence limit. The dual prediction system of PrDOS led us to choose this method since we consider that the use of templates guarantees a better estimation of this structural parameter. However, we evaluate other predictors, such as PONDR (http://www.pondr.com/) and IUPred3 (https://iupred.elte.hu/) which are based on the estimation of interactions between residues and hydrophobicity scores. In any case, although these predictors showed good performance and the general estimates for each sequence were quite similar, we decided to use PrDOS, considering it more accurate. In any case, for each studied sequence, predicted disordered were aligned residue by residue with the corresponding flexibility profiles.

## Results

### The π-helical conformation in S6 segment in TRP channels is associated with low local flexibility and low disorder

**Figure 1.**
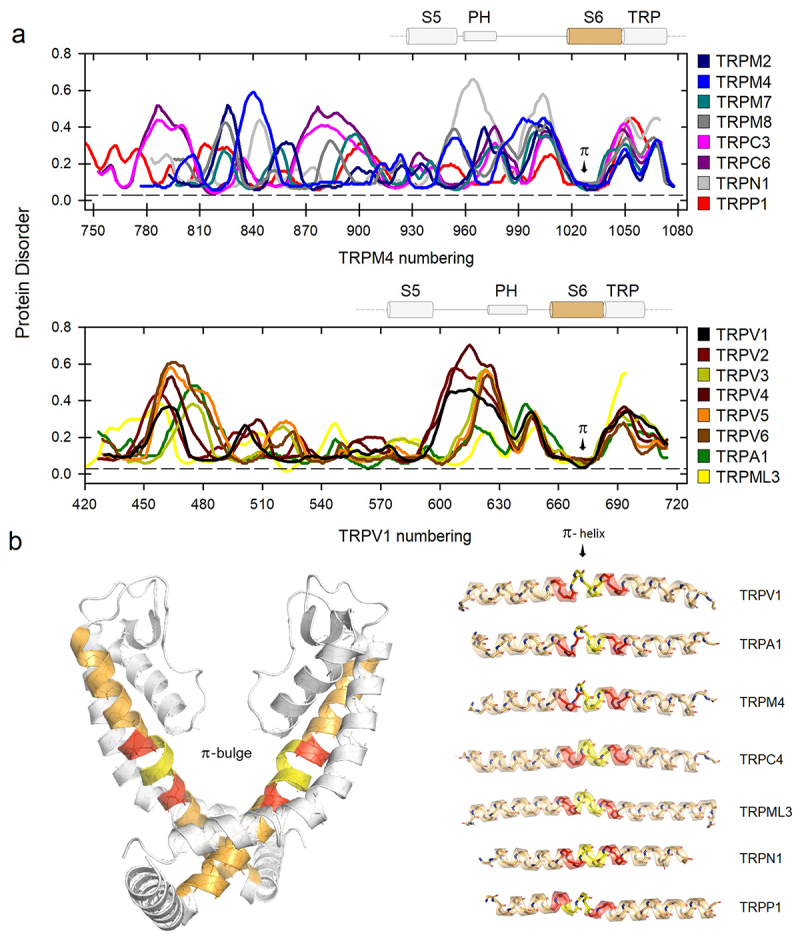
a) Disorder propensities for amino acid sequences of the VSLD in TRP channels. The region of higher protein order corresponds to the π-bulge at S6 (arrow). π-helices are depicted in yellow and α-helices in orange and red. b) Flexibility profiles were calculated based on the 11-residue sequence shown here in the red-yellow-red pattern. As an example, the structure of TRPV6 (PDB code 6bo8) shows the transmembrane pore helices bundle. The channel is oriented with the extracellular side on top. Two of the four monomers are shown for clarity. Only the S6 segment is presented in color.

First, we located those regions that transition to π or 3_**10**_ substructures within the TRP superfamily. To do this, we classified each sequence into five groups based on its degree of similarity. With this first alignment, we calculated the flexibilities associated with the π segments, which were essentially located in the middle part of the S6 segment, while the 3_**10**_ helices were essentially located in the C-part of S4. In this first phase of the analysis, the π regions found in some pore helices (PH) were not considered for the moment. However, the result of this analysis clearly shows that the flexibility profile of the helical segments associated with transitions to π-helices at S6 is significantly more rigid than those where the S4 segment transitions to 3_**10**_-helices (**Suppl. Fig. S1**, bar chart). In Figure 1, we also show that the so-called π-bulge is highly ordered in TRP channels. Likewise, structural evidence indicates that this conformation encompasses one helical turn (Figure 1b, segments in yellow), consistent with the typical configuration of the π-helix, which has 4.4 residues per turn and a pitch of 5.2 Å, making it wider and shorter than the canonical α-configuration [30].

With the aim of trying to reveal the local contribution of sequences neighboring this π-helix, we extended the analysis one turn before and one after this substructure (Figure 1B, segments in red), covering a total of 11 amino acid residues whose consensus is as follows:

**I**LTFV**LLLN**M**L** (mTRPV3) I663-L673

**I**IATL**L**M**LNLL** (hTRPV6) I564-L574

LVAN**ILL**V**NLL** (hTRPM4) L1029-L1039

LSTN**ILL**V**NLL** (mTRPM8) L965-L975

VTMVVV**LLN**M**L** (hTRPC3) V729-L739

**I**FVP**I**V**L**M**NLL** (hTRPA1) I946-L956

**Figure 2.**
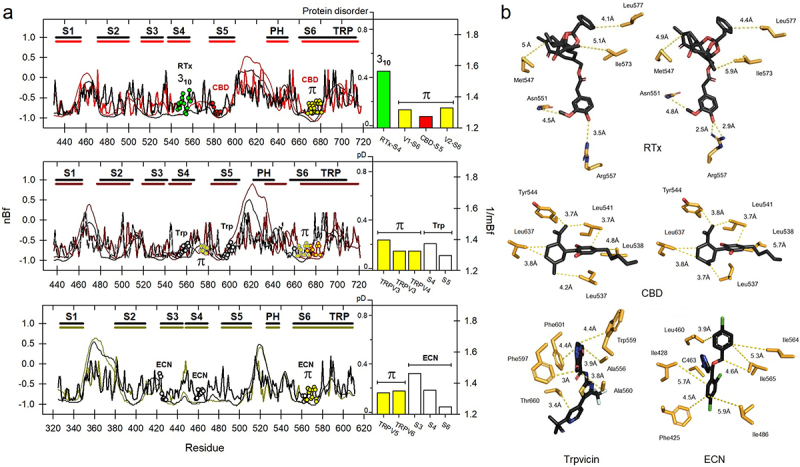
a) Normalized B-factors (nBf, thick lines) and protein disorder (pD, thin lines) for TRPV1–6 channels. Ligand binding sites and non-canonical substructures are indicated by circles (3_10_, green; π, yellow). Numbering is based on TRPV1, TRPV3 and TRPV5, respectively. The specific sequences of each segment can be consulted in Suppl. Fig. 1. Vertical bars show relative flexibilities calculated as the mean B-factor (1/mBf). B) Relevant interactions of RTx with TRPV1 (left: at 4°C, in a closed state, PDB code 7rqu; right: at 25°C, in an intermediate-open state, 7rqx); CBD-bound TRPV2 (states 1 and 2, PDB codes 6u8a and 6u88); human TRPV3 in complex with Trpvicin (7xj0) and TRPV5 from rabbit in complex with ECN (6b5v).

We then calculated the flexibility profiles for the six members of the vanilloid subfamily (TRPV) to compare them with the estimation of protein disorder. This analysis was performed by aligning residue by residue in parallel with the corresponding flexibility profile, overlapping both graphs. These results are depicted in Figure 2a. As can be deduced from these results, the external loop between S5 and the pore helix (PH), as well as the one between S1 and S2, are the most disordered and significantly flexible regions, as was originally suggested in cryo-electron microscopy studies [31]. This region, the so-called pore turret has been implicated in the allosteric coupling between the vanilloid binding pocket and the upper gate, regulating the mechanisms by which the channel opens and allows ion permeation [32]. The pore turret has also been implicated in the activation of those channels upon temperature increases and also determines ion selectivity [33,34]. These studies suggest that the pore turret could be very dynamic, contributing to the expansion of the upper gate. This region is significantly shorter in the TRPV3, V5, and V6 channels; however, the degree of protein disorder is still high in comparison. On the other hand, in these channels, the vanilloid compound binding site is configured in a pocket near the inner face of the channel, where part of S4 in TRPV1 participates in capsaicin and resiniferatoxin recognition [35]. Part of this binding site is configurated as a 3_**10**_ substructure that participates in the “head-down” accommodation of resiniferatoxin (RTx) with two residues, N551 and R557 (Figure 2b). Notably, the flexibility profile for this segment in S4 is significantly higher in comparison with the one for the π-bulge at the lower gate in S6, which is consistent with its participation in ligand accommodation events.

Since RTx binding in TRPV1 involves some residues that are configured in 3_**10**_ helices, we decided to explore this possibility in other members of the same subfamily and determine if other residues in non-canonical helical substructures also contribute to the accommodation of specific ligands. Such is the case of L637 in TRPV2, which participate as part of the binding site to cannabidiol (CBD). L537 and L541 on S5 also participate in the accommodation of this compound [36]. R539, on the other hand, in addition to being part of the CBD-binding pocket, also has been implicated in the binding to 2-APB [37]. In the case of TRPV1, there are several studies that confirm the fact that Y511, L515, M547, T550, L553, Y554, R557, E570, I573, and F587 have a role in the interaction with capsaicin and resiniferatoxin (RTx) [35,38,39]. With these references, we focused the flexibility analysis on two extra segments: A546–G558 for the S4 segment in TRPV1, and Leu537–Val546 for segment S5 in TRPV2. As can be expected, in Figure 2a (*upper* panel, bars), the flexibility profile for the binding site of CBD is similar to the one at the segment transiting from α-to-π (the lower gate at S6). On the other hand, the binding site for RTx, has higher mean flexibility (mBf_**S4b**_≈1.6), which is consistent with the 3_**10**_ helical conformation that the intracellular side of S4 (S4b) adopts in TRPV1 and with the heat-induced movement of S4b facilitating the loss of helicity of the C-terminal end of helix S4 [40]. Consistently with our hypothesis and based on the structure of human TRPV3 in presence of the agonist 2-APB, that protein also has a rigid π-helix at the S4–S5 linker (mBf_S4-S5L_≈1.3) which is critical during the ligand-dependent gating [41]. Our analysis shows that the central part of this linker (residues G573 to I579) is also significantly rigid (mBf_**S6**_≈1.4) (Figure 2a, *middle* panel, bars). This is also true in the case of binding to Trpvicin in TRPV3 and econazole (ECN) in TRPV5, where the associated flexibility profile is very similar, as it is part of an analog pocket [42,43]. In the case of ECN binding, both TRPV5 and TRPV6 some side chains that are part of regions that transit to π-helices with low flexibility profiles (mBf_**S3**_ = 1.46; mBf_**S4**_ = 1.36; mBf_**S6**_ = 1.25) are at optimal distances to facilitate this interaction (**Suppl. Fig. S2**).

The S4−S5 linker in the rabbit ortholog of TRPV2 also exhibit π-helices [44] which are intrinsically rigid (data not shown). In the case of the TRPV4 there are two available structures, one from *Xenopus tropicalis* and its human ortholog [45,46]. In both structures only the presence of a region 3_**10**_ located in the C-terminal part of S4 is evident for the amphibian protein. However, albeit the presence of π-helices in S6 has not been fully revealed in these cases, the available structural data for the human protein suggest that the translation of the S6 helix, hand in hand with the 1Å increase in the van der Waals radius, could be associated with the change in the registration of the side-chains of amino acids I715 and M718, which could indicate of this kind of helical transition [46].

**Figure 3.**
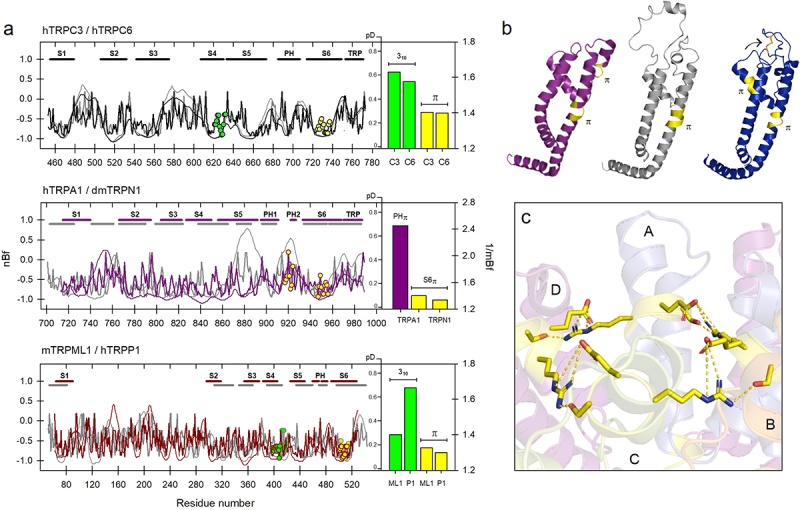
a) Flexibility profiles (nBf, thick), protein disorder (pD, thin) and mean flexibility (1/mBf) of non-canonical helical segments in representative members of the TRPC, TRPA, TRPN, TRPML, and TRPP families. Non-canonical substructures are indicated by circles (3_10_, green; π, yellow). Numbering is based on TRPC3, TRPA1 and TRPML1 proteins, respectively. The pore region in TRPA1 shows high flexibility and is associated with high disorder. b) S5-TRP segments in TRPA1 (3j9p, purple), TRPN1 (5vkq, gray), and TRPM4 (6bqr, blue) showing π-configurations (yellow) and pore loops. A disulfide bridge in TRPM4 (arrow) is implicated in the stabilization of the channel in the calcium-free state. c) Although configured in a disordered and flexible region, the π-helix present in PH2 of TRPA1 (yellow) is stabilized by a ring of salt bridges between R919/S921. All four participating monomers (A to D) are included.

Once the flexibility profiles for each member of the vanilloid TRP subfamily were studied, we decided to explore each subfamily under the same optics. To do this, we grouped the rest of the subfamilies following the criteria of phylogenetic similarity based on structural maps [47]. In this way, we grouped the canonical family (TRPC) separately from the ankyrin (TRPA1/TRPN1), melastatin (TRPM), and mucolipin/polycystin (TRPML/TRPP) families. In Figure 3A, six flexibility profiles for selected proteins (TRPC3/6, TRPA1, TRPN1, TRPML1, and TRPP1) are shown in detail. In all cases, the flexibilities were calculated for the ubiquitous π-helix in the upper region of S6 which acts as a hinge for these channels [48–52]. Likewise, the 3_**10**_-helical regions described for the TRPC3/4, TRPML1, and TRPP1 proteins in S4 were also estimated. As indicated in the corresponding plots, and as was previously depicted for the TRPV subfamily, those regions which transit to π-configurations are significantly more rigid than those transiting to 3_**10**_-helices.

Regarding TRPA1, it contains two pore helices, the second of which also has a π-helical twist [53]. We were surprised by the high flexibility of the 8-residue region part of PH2 in TRPA1 (N917 to E924), which has an mBf of 2.4, and is in a significantly disordered region (pD_**PH2**_ = 0.4) (Figure 3a, *middle* panel, bars). For this channel, as well as in the case of TRPN1 (NOMPC), no 3_**10**_-configurations were found within the transmembrane domain (the voltage sensor-like domain, VSLD). However, the π-substructure in PH2 of TRPA1, being in a highly disordered zone, is even more flexible than any of the regions which transit to 3_**10**_ helices studied in this work (*see* next section). In any case, the π-helix in PH2 of TRPA1 seems to be quite flexible as it appears as part of a highly disordered loop (Figure 3b).

In the next section we will describe this same pattern for members of the melastatin (TRPM) family, that is, segments at the middle part of S6, which are implicated in α-to-π-helical transitions have low flexibility profiles in comparison with those transiting to 3_**10**_-configurations. In sum, our analysis indicates that α-to-π helical transitions described for the middle part of segment S6, with an important role as the lower gate in TRPV, TRPC, TRPA1, TRPN1, TRPML1, and TRPP1 channels, as well as those were π-helices are present in the S4–S5L, are associated with low flexibility profiles and low disorder.

### Regions transiting from α-to-3_10_ helical substructures are associated with high flexibility profiles

**Figure 4.**
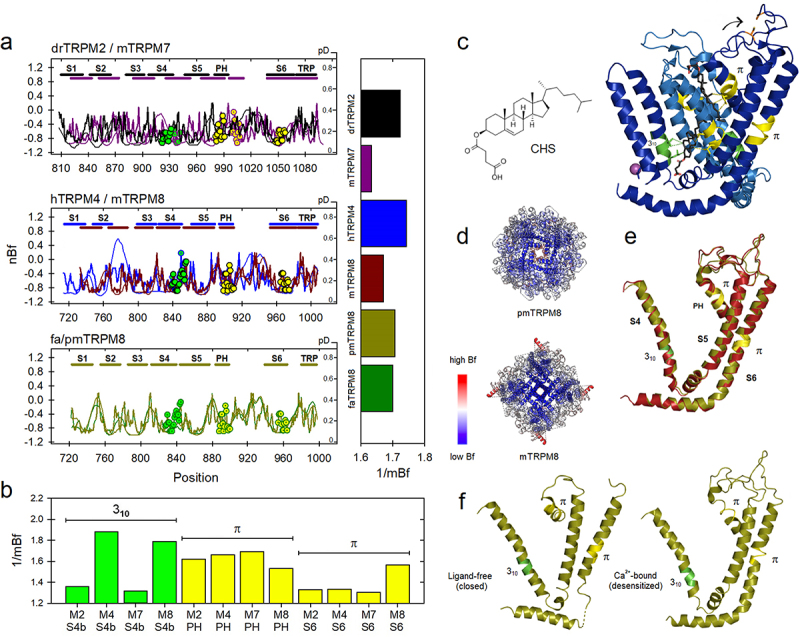
a) Flexibility profiles (thick) and protein disorder (thin) of non-canonical helical segments in representative members of the TRPM family. Non-canonical substructures are indicated by circles in green (3_10_) and yellow (π). Numbering is based on TRPM2, TRPM4, and avian TRPM8 proteins. Horizontal bars show the mean flexibility of the VSLD (including the TRP domain). b) Mean flexibility of 3_10_ and π segments. c) Ca^2+^-bound state of TRPM4 (6bqv) showing interaction with CHS and specific residues in non-canonical segments from two distinct subunits, calcium (sphere) and the breaking of the disulfide bridge at the pore loop (arrow). d) B-factor values colored from blue (low) to red (high) in avian (6o6a) and mammalian (7wra) TRPM8. As indicated by the mBf parameter for the transmembrane segment, the mouse TRPM8 channel is slightly stiffer in the pore region. E) Superposition of avian Ca^2+^-bound TRPM8 (olive, 6o77) and its ligand-free mammalian ortholog (red, 7wra); rmsd = 0.499Å. F) Disorder-to-order transition at the outer pore loop in pmTRPM8 upon calcium binding (6o77, right) and the equivalent closed structure in absence of Ca^2+^ (6o6a, left).

As we observed this correlation between regions of local rigidity favoring secondary transitions from α-to-π helices in TRP channels, we were wondering if α-to-3_**10**_ helical transitions are in some way also related to characteristic flexibilities. Following a global alignment of the TM-domain in the TRP superfamily, 3_**10**_ helices have been found in all subfamilies, particularly at the intracellular end of the S4 helices, except in TRPA1 and TRPN1 [14]. This expanded helix is equivalent to what has been observed also in voltage-gated ion channels [13]. In TRPV1, the 3_**10**_ regions at S4 are part of the rearrangements in the vanilloid pocket driving to the open state as a response to changes in temperature and capsaicin binding [54]; in TRPM8 an α-to-3_**10**_ transition is associated with icilin/PIP2 binding [55]. Based on this evidence, we calculate the local flexibilities for this region, where helix elongation occurs (the C-terminal part of S4, S4b), and find a comparatively high flexibility profile (mBf_**S4b**_ = 1.79). In TRPM4 this region has even higher intrinsic flexibility (mBf_**S4b**_ = 1.88). These data are shown in Figure 4, where it is also shown that 3_**10**_ regions in S4 are comparatively more flexible than those transiting to π-helices in S6 of TRPM2, M4, M7, and M8 channels. As it was previously demonstrated, this same trend was observed in the TRPV, TRPML, and TRPP families, and to a lesser extent in the TRPC family (Bar chart in **Suppl. Fig. S1**).

In TRPM8, three distinct groups have demonstrated structural rearrangements associated with the α-to-3_**10**_ conversion in segment S4b [55–58]. In that protein, π-helices in both the PH and S6 segment have also been identified during the transit from closed (antagonist bound) to the desensitized (Ca**2+** bound) state [57]. In Figure 4c we also confirm that, as in the case of TRPV5 (**Suppl. Fig. S2**), some residues within the regions that facilitate non-canonical helical transitions (α-to-π and α-to-3_**10**_) the TRPM4 protein in its interaction with calcium and cholesteryl hemi-succinate (CHS) could participate in the binding to this organic ligand: V904/L907 (3_**10**_ region) and V1030/L1034 (π region). These residues are located in two neighboring subunits [59]. Notably, the π regions in S6 of TRPM8 are significantly more flexible than π regions in TRPV channels (compare bar charts in Figures 2a and 4), which is consistent with the greater flexibility that the TRPM family has with respect to TRPV, at least in the transmembrane region [60]. As we reported in that study, the variability in flexibility profiles within the TRPM family is high and this supports the fact that the three TRPM8 orthologs whose structure is now known (*F. albicollis*, *P. major*, and mouse) show different global flexibilities, being the mammalian ortholog significantly more rigid than the one present in birds (compare for example horizontal bars in Figure 4a with the global flexibility of each structure in terms of the B-factor in Figure 4d). Remarkably, even being slightly more flexible than the mammalian ortholog, the external structure of the outer pore in *P. major* (pmTRPM8) is ordered almost identically to mTRPM8 (rmsd = 0.5Å). This only occurs in presence of calcium, when the channel is stabilized in a desensitized state by inducing large structural rearrangements at the pore loop (Figure 4e) [57].

The sequence analysis we performed on TRP proteins prompted us to check if α-to-3_**10**_ helical transitions have been also characterized in other channels as the phylogenetically distant voltage-dependent Kv channels. The VSLD in TRPM and the VSD in Kv1–4 channels were also recently reported by us as highly flexible in the voltage-gated ion channel (VGIC) superfamily [60]. Transitions to 3_**10**_ conformations facilitate the downward motion of segment S4 during the gating process in the so-called *paddle* chimera (Kv1.2–2.1) [61]. Although this is a synthetic construct, it is interesting to explore such S4b reconfiguration exclusively in terms of amino acid composition, as this chimera has the Kv2.1 S3b-S4 region inserted within the Kv1.2 channel sequence [62]. Indeed, this transition has been reported in several K- and Na-channels [13]. In the Shaker Kv channel, a short-lived 3_**10**_-helical structure has been well identified at the S4b segment during channel activation [63,64]. Experimental evidence also indicates that although this part of the protein in KCNQ1 channels (Kv7.1) is mainly α-helical, it has the potential to transit to 3_**10**_ configurations depending on the milieu conditions [65]. The prevalence of this helical element in Kv, Nav, HCN, and TRP channels could be unique to the design plan of the VGIC superfamily. In a study where more than 230 protein structures were analyzed, it was determined that the 3_**10**_ helices are typical of potassium channels but also frequent in other membrane proteins (*e.g*. in cytochrome bc(1) or cyanobacterial photosystem II complexes) and that the length of this substructure could depend on its interaction with membrane lipids (13).

**Figure 5.**
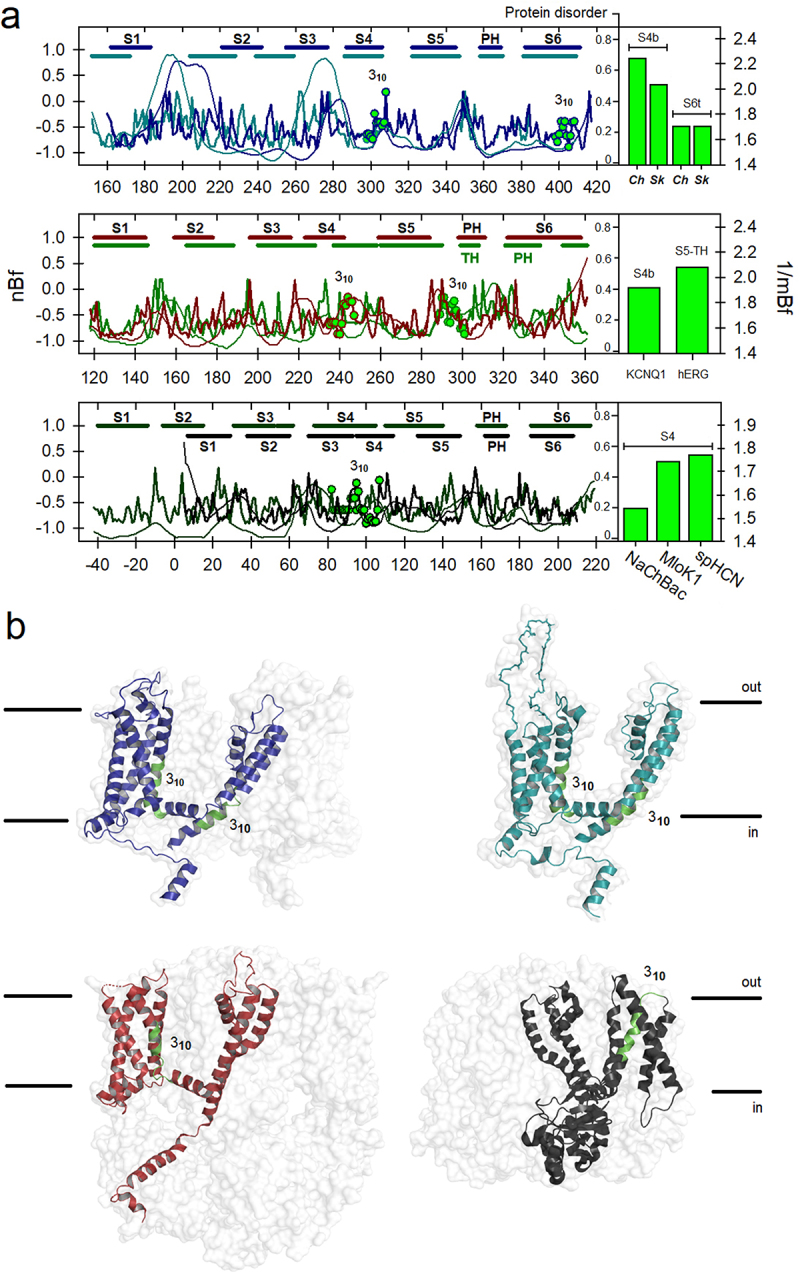
a) Flexibility profiles (thick) and protein disorder (thin) of S1-S6 segments in representative members of the VGIC superfamily. Regions prone to achieve 3_10_ helical configurations are depicted as green circles. In descending order, the profiles are shown overlapping in pairs: the Kv1.2–2.1 chimera (dark blue) and the Shaker-E channel (dark cyan); the KCNQ1 (dark red) and hERG (dark green) channels, and the bacterial MloK1 (black) and spHCN (forest green) channels. The numbering corresponds to that of the first member in each pair. Vertical bars show relative flexibilities for segments involved in α-to-3_10_ helical transitions as calculated before. The nBf profile and pD plot for NaChBac is not shown for clarity. B) Physical location of flexible regions facilitating α-to-3_10_ helical transitions in the paddle chimera (dark blue, 6ebm), Shaker-like Kv1.2 (dark cyan, 3lut), Kv7.1 (dark red, 6uzz), and MloK1 (black, 4chv). Membrane limits are indicated.

The possible implication of the 3_**10**_ configuration in the motion of the lower gate during activation has been also suggested by MD simulations [66]. In hERG channels, a very dynamic S5-PH segment (residues 577–583), transitions to 3_**10**_ helical conformations [67]. Our analysis shows that this region in that protein is slightly disordered and flexible (Figure 5A, *middle* panel). We also include MloK1, NaChBac, and spHCN channels in the analysis since the presence of this helical configuration in those channels [68–70]. The estimated flexibilities indicate that these segments adopting 3_**10**_ configurations in S4b, S5-TH, and S6t are even more flexible than the S4–3_**10**_ regions of TRP channels, except in the case of the segment S4 of the NaChBac channel (mBf = 1.54; Figure 5a, *lower* panel, bars). The estimated mBf for the S4b segment were: 2.24 (*paddle* chimera), 2.03 (Shaker-E), and 1.92 (Kv7.1). On the other hand, S4 flexibility in MloK1 has an mBf of 1.74, 1.77 in spHCN, and 1.8 in HCN1. The mBf for the sequence involved in α-to-3_**10**_ transitions into the S5-PH linker in hERG channel (residues M574 to L586) is 2.08. Regarding local flexibility for S6t in the *paddle* chimera and Shaker-E, it is also quite high (mBf≈1.7) in both cases, due to the high sequence identity in these two proteins. As can be deduced, these values are in fact like those predicted for the analyzed segment S4 in TRPM4/M8 (compare vertical bars in Figure 4b).

The flexibility profile for segment S4b in the Shaker-E channel was however not as high as the one for the Kv 2.1–1.2 chimera. This probably is because the chimera has a Leu at position 298 and Lys at 302 (K5 in the nomenclature of S4 charges). Isoform E, on the other hand, has Phe and Arg residues in the equivalent positions. Leu and Lys side-chains are significantly more flexible than those of Phe and Arg [23]. In any case, it makes sense that the S3–S4 linker in this isoform is at least three times longer, suggesting that the flexibility needed to favor the local motion of segment S4 during channel activation, in addition to requiring the facilitation derived from the α-to-3_**10**_ transition, is in part also accomplished by this long linker, which is quite disordered and highly flexible (Figure 5b).

### Local flexibility and protein disorder are related but not equivalent parameters, exhibiting significant discrepancies in dynamic regions

Protein flexibility is related to protein disorder, but they are two distinct concepts. Whereas protein flexibility refers to the intrinsic degrees of freedom along the polypeptide chain, mainly as a function of the steric and charge contributions, as well as the exposure to the solvent for each amino acid side-chain [71,72], protein disorder alludes to the lack of stable secondary structures. Furthermore, this last parameter is based primarily on H-bonding patterns as well as geometric constraints [71]. In that sense, protein disorder concerns the absence of structural constraints, mainly electrostatic in nature, and consequently, to an inherent conformational heterogeneity [73,74]. In Kv and TRP channels, outer pore loops or “turrets” are disordered regions of great importance in terms of function. For example, they participate in modulation events by extracellular divalent cations, to stabilize the fully open state, or as part of the temperature-sensing machinery [33,75,76]. In general, extramembrane loops are also known to influence the tertiary structure and stability of membrane proteins in terms of the distribution of hydrophobic residues, which affects protein function [77]. With these precedents, after having studied the co-dependence of intrinsic flexibility in ordered regions and the propensity to reconfigure α-helices to π or 3_**10**_ substructures, we then focus on studying unstructured (disordered) regions. Our rationale was that given the well-documented physiological relevance of these regions, finding some relationship between their degree of disorder and intrinsic flexibility could reveal important dynamic information.

The result of this analysis shows a correspondence between these two parameters, that is, in general terms those regions of high flexibility are generally disordered regions and vice versa. Interhelical linker regions (loops) exhibit a considerable degree of disorder, which is directly proportional to the linker length (**Suppl. Fig. S3**). In thermo-TRP channels TRPV1–4 the region of greatest disorder is the one connecting the S5 helix to the PH, the so-called extracellular large pore turret [32], a subdomain that faces the lipid-water interface, and stabilizes the reentrant pore helix [78]. In the case of TRPM8, this is not the case since the region with the highest degree of disorder is the outer pore loop (PH-S6L) [57]. In Kv Shaker-like channels, the linkers between segments S1 and S2 (S1–S2L) as well as the one between segments S3 and S4 (S3–S4L, included in the so-called *paddle* motif) are those with a high degree of disorder. The pore turret loop also shows this trend, although to a lesser extent in all protein structures analyzed (compare thin lines in Figures 2 to Figure 5).

**Figure 6.**
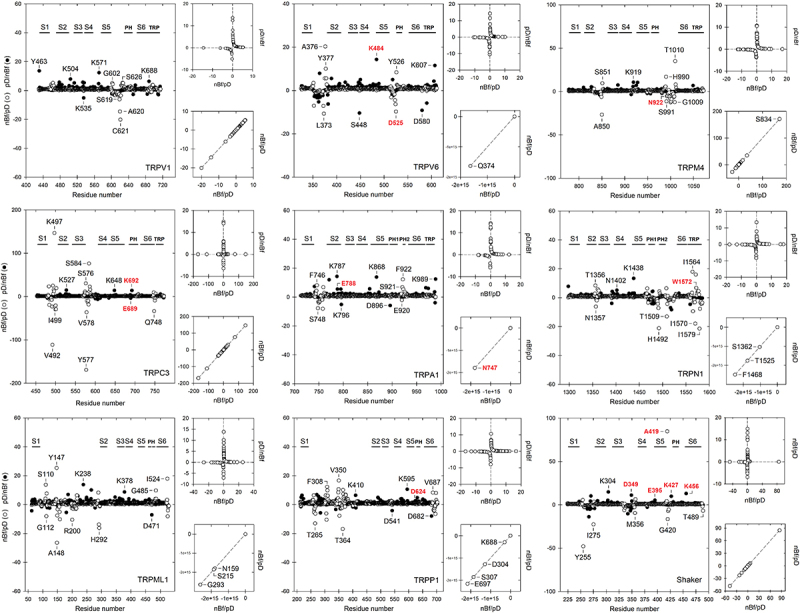
Relationship between the normalized B-factor (nBf) and the protein disorder index (pD) for each residue of the transmembrane domain in selected members of the TRP superfamily and the Shaker Kv channel. White circles represent the flexibility parameter as a function of its degree of order, while the black ones indicate the opposite quotient. Both ratios allow highlighting residues that show some remarkable discrepancy between these two parameters. Residues that exhibit this kind of structural discrepancy are shown as one letter codes, whereas residues in red are those whose relevance in structural, dynamic, or functional terms have been experimentally confirmed (see Table 1).

A more exhaustive analysis of each residue in these regions indicates that a high disorder is not necessarily composed of residues considered as flexible, but that in certain positions a sequence with a high degree of protein disorder can be composed of rigid residues. Hence, a certain degree of discrepancy between the flexibility and disorder is outstanding where these loops are found (Figure 6). These data, in addition to confirming that the extracellular regions are highly disordered, are regions where rigid and frequently hydrophobic residues could favor protein disorder [23]. Nevertheless, many TRP structures exhibit these regions folded (see for example the pore loops in TRPA1, TRPN1, and TRPM4 in Figure 3B). This would indicate that those regions are stabilized in some way, probably upon ligand binding events. In that sense, these disordered loops have a high degree of conformational heterogeneity and consequently could be very dynamic, acquiring a more stable tertiary structure under certain conditions, such as the allosteric effects in ligand interactions [79]. In sum, flexibility and disorder are not synonymous: a flexible region could favor a stable structure (ordered), and vice-versa. The relevance of this observation from the perspective of the physiological role that has been discovered for extracellular regions in integral membrane proteins could be quite significant.

**Table 1.** Data dispersion between the flexibility parameter (nBf) and the disorder parameter (pD).

	S.D. (σ) nBf/pD	S.E.M.	Relevant Residues^a^	References
TRPV1	1.990	0.118	P608, P623, N628	[91,92]
TRPV6	2.003	0.118	D525, K484, R589	[12,93]
TRPM4	10.551	0.614	N992	[94]
TRPC3	15.799	0.882	E689, K692	[95]
TRPA1	1.468	0.088	N747, I751, I752, E788, R919	[96–98]
TRPN1	3.215	0.190	W1572	[50]
TRPML1	2.717	0.126	D111, D114	[99]
TRPP1	2.221	0.101	D624, W414	[100]
*Shaker*	6.375	0.391	D349, E418, A419, K427, K456	[76,101]

^a^Residues with structural discrepancy that have been mutagenized and reveal important functional significance are shown.

Results shown in Figure 6 indicate that some residues with this type of structural discrepancy could be of great functional relevance. As expected, the degree of discrepancy between the predictive parameter of intrinsic flexibility (nBf) and the structural parameter of disorder (pD) is always dependent on the context of each protein but is regularly associated with the linker regions of each channel. Finally, in the case of the TRPV6, TRPM4, TRPC3, TRPA1, TRPN1, TRPP1, and Shaker channels, residues with significant structural discrepancy (highlighted in red) are generally associated with glycosylation sites, allosteric regulation, or are dynamic in terms of protein activation (Table I).

## Discussion

To investigate the relationship between flexibility, protein disorder, and the propensity to acquire non-canonical helical configurations in specific segments of TRP and Kv channels, we focused on two basic questions. First, since non-canonical helical transitions are relatively common in proteins because these biomolecules are highly dynamic entities [80], and since such substructures might be associated with composition [81–86], the helical transitions reported during TRP/Kv/HCN activation are predetermined in some way in their sequences and in turn with a characteristic flexibility index? Second, what relevant information – in functional terms – could be extracted from contrasting these two apparently co-related predictive parameters (i.e. side-chain flexibility and protein disorder)?.

In this work, we answered the first question in the affirmative, at least in the studied channels: those regions that transition to expanded non-canonical helical substructures (3_**10**_) are preferentially associated with amino acid sequences with high flexibility profiles, while those that transit to π-configurations are in areas of significant rigidity. However, in the latter case, we find that certain regions of high flexibility (for example the pore helix in some TRP channels) are also prone to transition to π-configurations. In such situations, the evidence we found suggests that these substructures acquire a certain degree of structural stabilization through additional elements such as the formation of a sort of salt bridges ring, as in the outer pore of TRPA1 [53] (Figure 3C) or the presence of a possible disulfide bond in the turret of TRPM4 [87] (Figures 3B and 4c).

In this context, we believe that although the flexibility of a given segment depends primarily on its amino acid sequence, other factors such as this class of atomic interactions, the exposure to the solvent, or the binding of specific ligands must play a pivotal role in the formation and stabilization of such structures and so determine specific functional states. Even so, our compositional and structural analysis clearly indicates that despite these remarkable peculiarities, the 3_**10**_-regions are more flexible than the π-regions in S6 or even the PH in TRP, Kv, HCN, and Nav channels (**Suppl. Fig. S4**). Hence, although the presence of a non-canonical helical substructure could be mainly determined by the local flexibility of the segment in question, flexibility itself is not the only factor determining such transitions.

Regarding the second question, we have submitted to scrutiny two structural parameters that, contrasted with each other, have revealed residues whose relevance in functional terms could be highlighted. At this point, we can establish that the flexibility and the degree of disorder in a protein are not equivalent parameters, although they could be closely related. Protein flexibility has many meanings and attributes; here we refer to the conformational aspect of this parameter. In that sense, intrinsic flexibility is related to the number and geometry of amino acid conformers, their stability, the associated energy barriers between them, and more specifically, the atomic kinetic parameter or thermal motion (B-factor) [1,71,80]. Protein disorder, instead, refers to regions that lack a stable three-dimensional structure (secondary or tertiary), and functional; it also refers to the spatial location of amino acid residues, as well as the multiple conformations they acquire and which can rapidly interconvert in a time-dependent way [29]. This being the case, it is not surprising that from a non-expert view, both parameters are frequently confused or misinterpreted. However, there is a persistent relationship that is pretty obvious: flexible regions, *i.e*. regions with high B-factor, are regions that frequently tend to be disordered and vice-versa [24]. Thus, determinants for flexible and intrinsically disordered regions could be directly correlated with the primary sequence.

It has been the subject of intense debate trying to answer the question of whether flexibility and pD are predetermined by the primary sequence; thus, significant efforts have been made to address this dilemma [22–24]. In terms of composition, pD indicates the lack of bulky hydrophobic amino acids, having as consequence being unable to form stable hydrophobic regions and, in consequence, structured domains [29]. On the other hand, although flexible regions tend to be disordered, they can be ordered through electrostatic interactions that stabilize them [24]. In this context, some reports indicate that π-helices are especially rich in hydrophobic – frequently aromatic – amino acids (W, F, Y, L, I, M, and V with a preference to include Asn in the middle position in short (up to 7 residues) segments of proteins [18,84]. This is consistent with our results since the π-bulge at the lower gate in segment S6 is highly ordered in TRP channels and a very conserved Asn residue is completely conserved in practically all these sequences (**Suppl. Fig. S1**). Indeed, N676 in TRPV1 participates in the formation of the so-called π-bulge [88]. The preference for P, K, and G at the C-cap position in π-helices has also been noted, while Asp and Thr are more frequent at the N-cap position [84]. This compositional preference could indicate a series of attractive van der Waals interactions between side-chains, which make such configurations highly metastable structures. This evidence could indicate that the “elusive” π-helix is not as unstable as it has been suggested [82]. Our data would confirm this because this helical configuration is associated with a certain stiffness. In any case, the distribution of amino acids in π-helices could be more complex than expected and probably difficult to predict due to its context dependence [85].

3_**10**_ helices have been described as amphipathic, with a noticeable propensity to find D, P, N, S, G, L, A, T, and, to a lesser extent, K and R (*i.e*. residues considered flexible). Here, we find that L, K, and R are in general present in those segments. The amphipathicity of this substructure is important for the stabilization of protein folding [19,86]. Given this evidence, it appears that, as in π-helices, there is no such thing as a consensus sequence or predictable motif. This would support the notion of using flexibility profiles as an additional criterion for the detection of these helical substructures. The 3_**10**_ configuration, which is the longest of the three substructures, is stabilized by intrachain H-bonding, cross-linking the C=O group of amino acid residue *i* with the NH group of residue *i* + 3 [72]. These helices have been preferentially observed at the termini of α-helices, are associated with regions with high Bf values, and compared to α- and π-helices, it is the most intrinsically flexible helical structure, which is consistent with the fact that these regions are found preferentially in areas close to the aqueous interface of the membrane (Figure 5B). This situation makes it an ephemeral structure, not very stable and indeed very dynamic as well as more “elusive”. In TRPM8 the α-to-3_**10**_ transition in S4 contributes to the reconfiguration of the S4−S5L in response to calcium binding [57] whereas, in typical voltage sensors, that conversion could facilitate the S4 motion during the transfer of gating charges across the electrical field in response to membrane potential [89].

We also discover that disordered regions are not always flexible. This confirms that we can’t judge a book by its cover. Our dual flexibility/disorder analysis provides evidence to help distinguish potentially important structural elements participating during the stabilization of different functional states of ion channels. In that sense, the case of the conformational transition of avian TRPM8 is of particular interest since the conformational changes associated with the binding of agonistic compounds have an important impact on the organization of extracellular disordered regions, *i.e*. this region undergoes a physiologically relevant disorder-to-order transition in response to ligand binding [55,57]. Therefore, we decided to further evaluate those regions that bind specific ligands and establish whether those regions which facilitate helical transitions are also involved in the interaction with specific ligands. Results in Figure 2, Figure 4, and **Suppl. Fig. S2** indicates that several residues which are part of the rigid (π) or flexible (3_**10**_) regions contribute to the potential stabilization of several ligands in TRP channels. Notably, some of them are part of the non-canonical regions and could be of great relevance in functional terms. Such is the case of R557 (3_**10**_) in TRPV1 interacting with RTx; L637 (π) in TRPV2 with CBD; I565(π) in TRPV5 with ECN; V465/A469 (3_**10**_) in TRPV6 with ECN, or L907 (3_**10**_) and V1030/L1034 (π) in TRPM4 in its interaction with CHS. Therefore, residues located in these non-canonical substructures can have important functional effects; such is the case of Y554 (part of a highly flexible substructure 3_**10**_ in S4), which has been reported as quite mobile during heat activation in TRPV1 [40]; I679 (near to the π region of S6) also contributes during the expansion of the lower gate as part of the heat activation mechanism in that channel [90]. A566 (part of the π-substructure in S6) collaborates in the pore expansion of the lower gate during the activation of TRPV6 [12]. In addition to these examples, as mentioned above, other residues could be of great functional relevance given their discrepancy in the two parameters analyzed here, *i.e*. flexibility and disorder (Figure 6, Table 1).

To conclude, flexibility in proteins depends on many factors but it is mainly determined by the composition of amino acids. This parameter, strongly dependent on the degrees of freedom of the side-chains, could configure a local segment within a protein to behave dynamically or statically in terms of the degree of order they acquire. From a functional perspective, the role of interaction with diverse chemical ligands or how a change in the membrane potential facilitates helical reconfigurations and disorder-to-order conformational transitions is of great relevance to understanding why these proteins do what they do.

## Supplementary Material

Supplemental Material

## Data Availability

Data supporting the findings of this study are openly available by direct contact to the corresponding author.

## Abbreviations

2-APB, 2-aminoethoxydiphenil borate; CBD, cannabidiol; CHS, cholesteryl hemisuccinate; ECN, econazole; PIP2, Phosphatidylinositol 4,5-bisphosphate; RTx, resiniferatoxin; mBf, mean flexibility; nBf, normalized B-factor; pD, protein disorder.
