# Supplementary material for: Non-canonical helical transitions and conformational switching are associated with characteristic flexibility and disorder indices in TRP and Kv channels
Source: Channels (Austin). 2023 May 17;17(1):2212349. doi: 10.1080/19336950.2023.2212349 (PMC10193913; doi:10.1080/19336950.2023.2212349)
Supplement: Supplemental Material [file KCHL_A_2212349_SM1193.zip › Supplementary files/Supplementary caption.docx]

**Supp. Fig. 1.** Multiple sequence alignment and sequence logos per-position of the TRP superfamily. The Clustal-Ω alignment indicates the degree of sequence conservation in segments S4 to S6 for each analyzed member. Non-canonical helical conformations are depicted in green (3**_10_**) and yellow (π). Bars represent the distribution of the mean B-factor in those non-canonical segments inside segments S4 (3**_10_**) and S6 (π). S.D. is included.

**Supp. Fig. 2.** Ribbon diagrams of TRPV5 (6b5v) and TRPV6 (7s8c) from rabbit and human respectively. The segments involved in the interaction with ECN are shown, particularly those residues which favor non-canonical helical transitions in green (3**_10_**) and yellow (π). In both cases the interatomic distances shown oscillate between 4.4 and 5.4 Å.

**Supp. Fig. 3.** Protein disorder of external loops as a function of the linker length. These dependencies are shown for the six S3-S4 linkers present in the VGICs studied (dark cyan), for the S5-PH pore loop (turret) of the TRPV1-6, TRPC3 and C6, TRPA1, TRPN1, TRPML1, and TRPP1 (dark red), as well as for the PH-S6 loop in TRPM2, M4, M7, and M8 (blue).

**Supp. Fig. 4. A**) Comparison of mean flexibilities in the analyzed 3**_10_** and π substructures. 18 3**_10_** substructures in TRP (V1, C3, C6, ML1, P1, P1, M2, M4, M7, M8) and VGIC (paddle chimera, Shaker-E, KCNQ1, hERG, MloK1, NaChBac, spHCN) and 22 π substructures in TRP channels (V1-6, C3, C6, A1, N1, ML1, P1, M2, M4, M7, M8) are included. The error bars represent standard deviations. **B**) Distribution of five (*i* + 4) residues on two representative non-canonical helices. 3**_10_**-helix (green) and π-helix (yellow) are displayed in side-view. The PDB ID are given below each representative example.
