# Supplementary figures and images for: Non-canonical helical transitions and conformational switching are associated with characteristic flexibility and disorder indices in TRP and Kv channels
Source: Channels (Austin). 2023 May 17;17(1):2212349. doi: 10.1080/19336950.2023.2212349 (PMC10193913; doi:10.1080/19336950.2023.2212349)

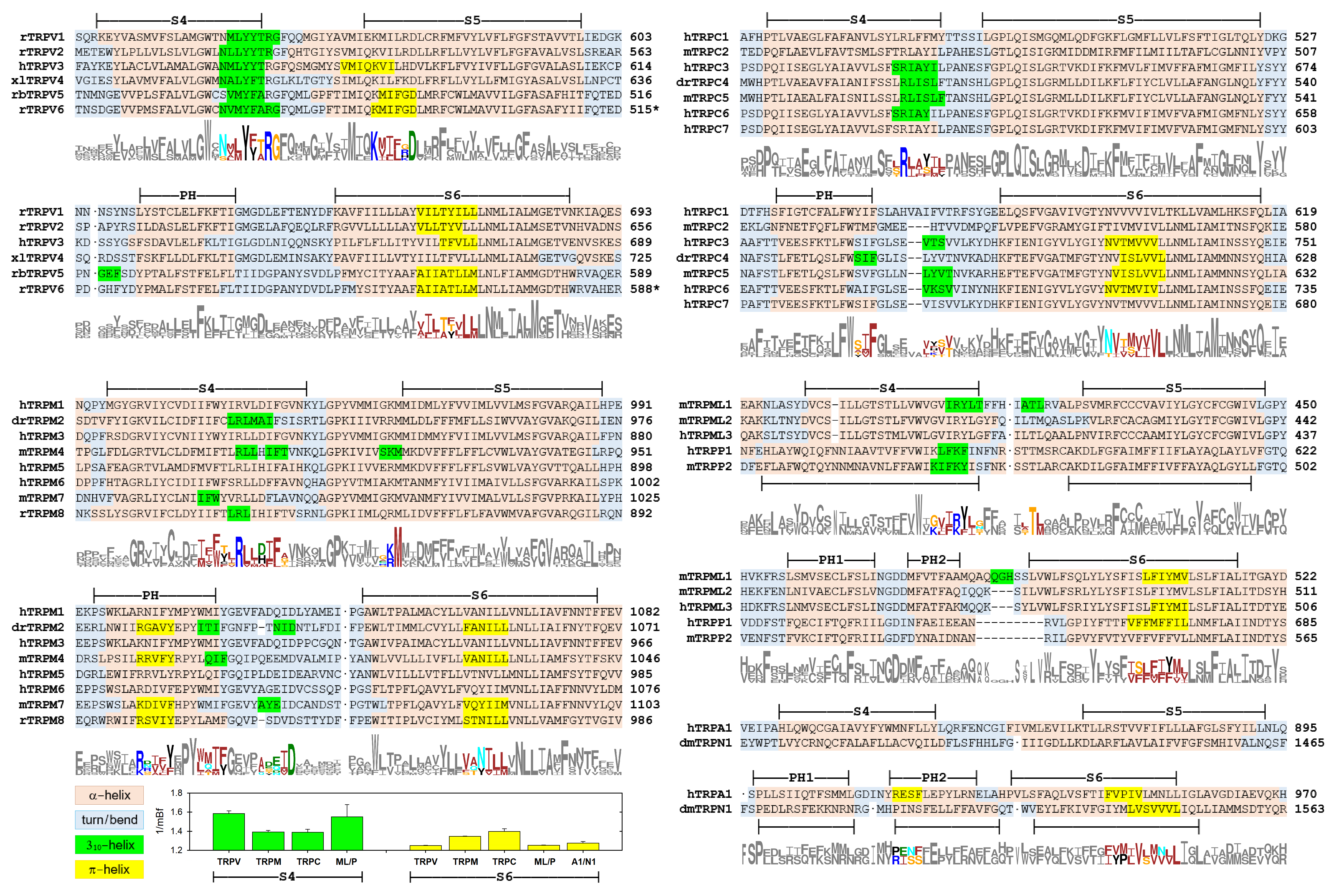

Supplement: Supplemental Material [file KCHL_A_2212349_SM1193.zip › Supplementary files/FigureSupp_1.tiff]

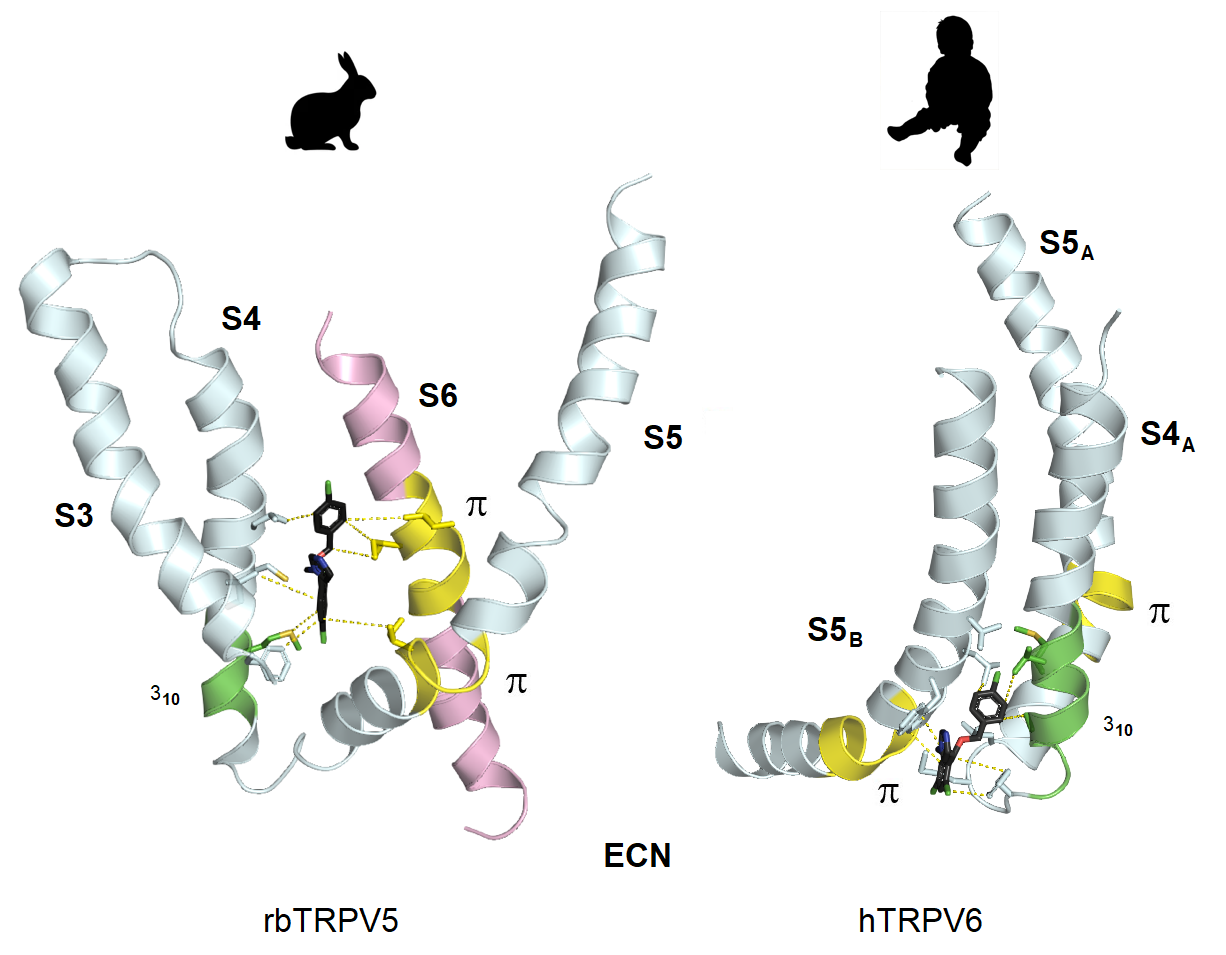

Supplement: Supplemental Material [file KCHL_A_2212349_SM1193.zip › Supplementary files/FigureSupp_2.tiff]

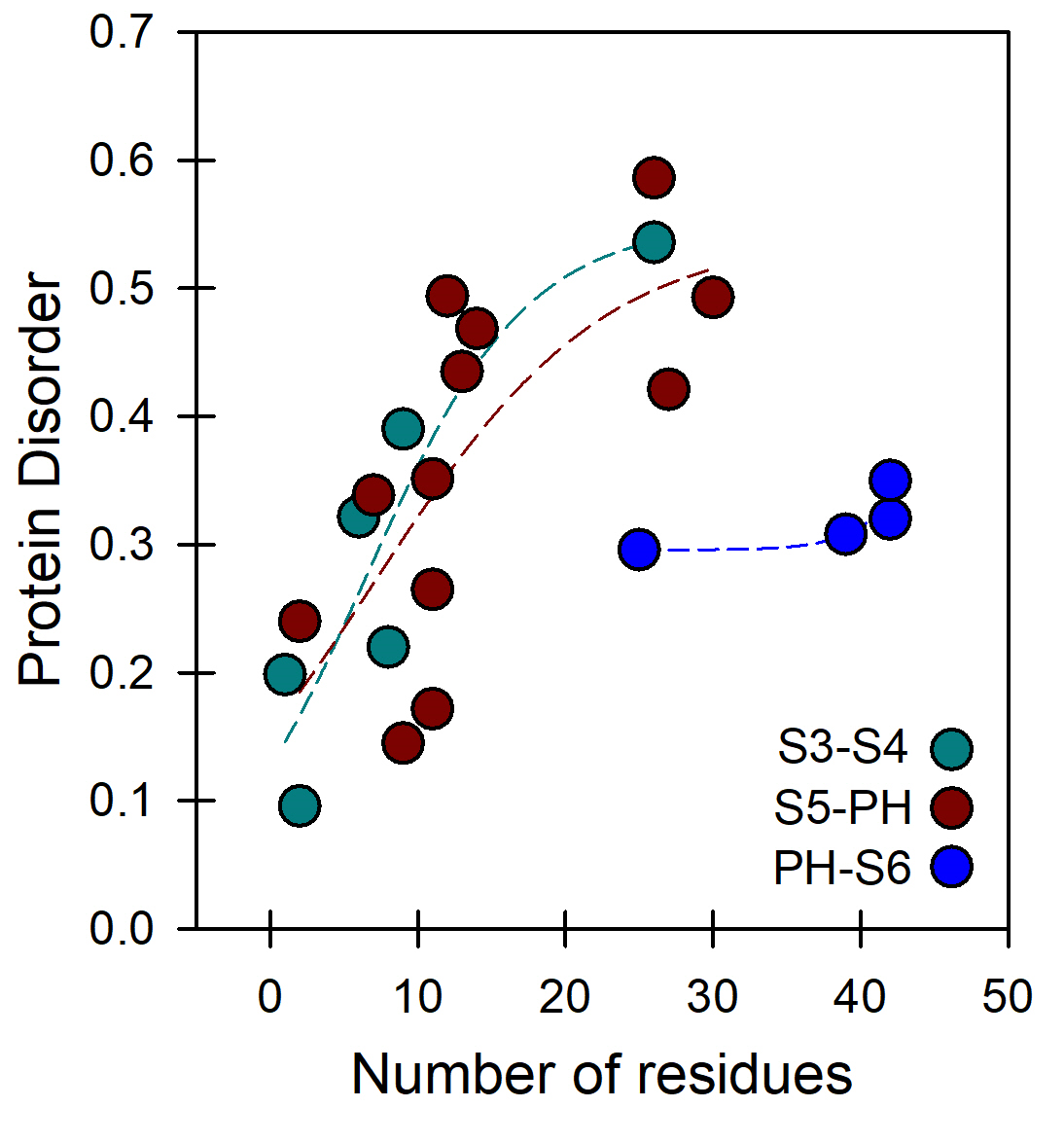

Supplement: Supplemental Material [file KCHL_A_2212349_SM1193.zip › Supplementary files/FigureSupp_3.tiff]

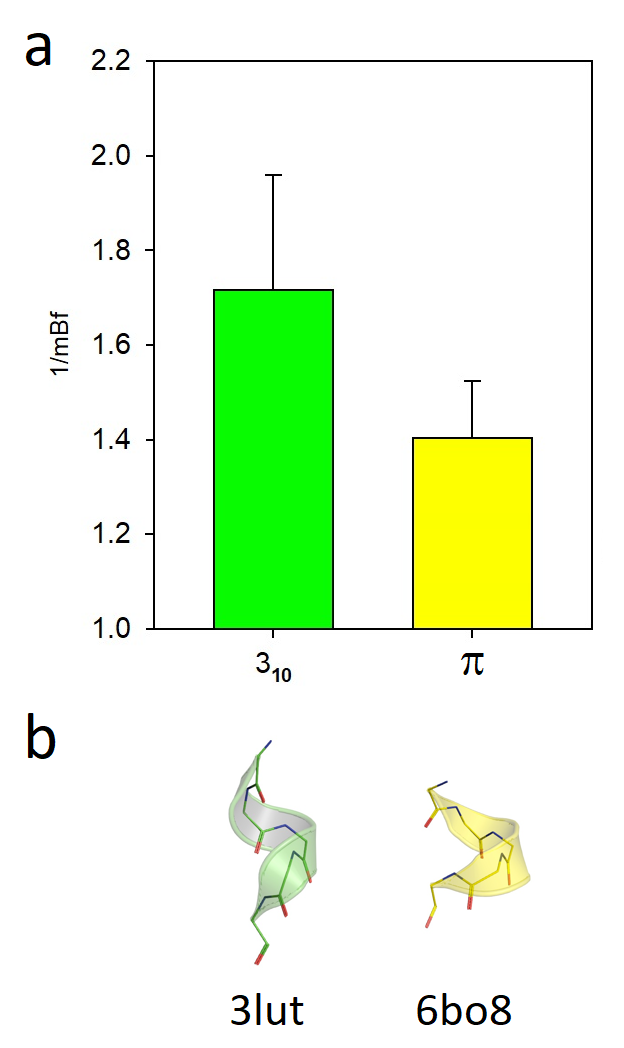

Supplement: Supplemental Material [file KCHL_A_2212349_SM1193.zip › Supplementary files/FigureSupp_4.tiff]
